# An Evaluation of the Dementia Friends USA Program in Nevada: Changes in Knowledge and Behavioral Intent

**DOI:** 10.1093/geroni/igaa057.092

**Published:** 2020-12-16

**Authors:** Jennifer Carson, Peter Reed, Zebbedia Gibb

**Affiliations:** 1 University of Nevada, Reno, Reno, Nevada, United States; 2 University of Nevada, Reno School of Medicine, Reno, Nevada, United States

## Abstract

Dementia Friendly Nevada (DFNV) aims to develop and promote communities in becoming more respectful, educated, supportive and inclusive of people living with dementia and their care partners. To date, six communities are engaged, representing urban, rural and tribal communities. Each community convened an action group comprised of volunteers from a range of sectors, including people living with dementia as key participants. Each group used a participatory action research process to assess community needs, develop specific goals and actions, and document community change. As part of this process, five of the six communities adopted the Dementia Friends program for community education and awareness raising. This program is licensed nationally by Dementia Friends USA. From June 2018 – September 2019, these communities achieved widespread dissemination of this program, training 68 Dementia Friends Champions (i.e., trainers), who in turn delivered 55 training sessions to 607 new Dementia Friends (i.e., completers) across Nevada. To evaluate the impact of the program, DFNV partnered with the Sanford Center for Aging to conduct a pre-post survey (n = 504) to assess program-related knowledge change as well as commitments to take action. Results showed a statistically-significant (p <.001) increase in participant knowledge, with 17% higher scores at the post-assessment. In addition, given 10 options for specific actions, responses ranged from 12% of participants committing to “start a dementia friendly effort” to 70% committing to “support dementia friendly efforts.” Overall, the early impact of this ongoing effort to educate the public about dementia across Nevada has been very successful.

